# The Association of Genetic Predisposition to Depressive Symptoms with Non-suicidal and Suicidal Self-Injuries

**DOI:** 10.1007/s10519-016-9809-z

**Published:** 2016-09-02

**Authors:** Dominique F. Maciejewski, Miguel E. Renteria, Abdel Abdellaoui, Sarah E. Medland, Lauren R. Few, Scott D. Gordon, Pamela A.F. Madden, Grant Montgomery, Timothy J. Trull, Andrew C. Heath, Dixie J. Statham, Nicholas G. Martin, Brendan P. Zietsch, Karin J.H. Verweij

**Affiliations:** 1 Department of Clinical Developmental Psychology and EMGO Institute for Health and Care Research, Vrije Universiteit Amsterdam, 1081 BT Amsterdam, The Netherlands; 2 Genetic Epidemiology, Molecular Epidemiology and Neurogenetics Laboratories, QIMR Berghofer Medical Research Institute, Brisbane, QLD 4006 Australia; 3 Department of Biological Psychology, Vrije Universiteit Amsterdam, Van der Boechorststraat 1, 1081 BT Amsterdam, The Netherlands; 4 Department of Psychiatry, Washington University School of Medicine, St Louis, MO 63110 USA; 5 Department of Psychological Sciences, University of Missouri, Columbia, MO 65211 USA; 6 Faculty of Arts and Social Sciences, University of Sunshine Coast, Sippy Downs, QLD 4556 Australia; 7 School of Psychology, University of Queensland, St. Lucia, Brisbane, QLD 4029 Australia

**Keywords:** Depression, Suicidal ideation, Suicide attempts, Self-injury, Polygenic risk, Genetics

## Abstract

Non-suicidal and suicidal self-injury are very destructive, yet surprisingly common behaviours. Depressed mood is a major risk factor for non-suicidal self-injury (NSSI), suicidal ideation and suicide attempts. We conducted a genetic risk prediction study to examine the polygenic overlap of depressive symptoms with lifetime NSSI, suicidal ideation, and suicide attempts in a sample of 6237 Australian adult twins and their family members (3740 females, mean age = 42.4 years). Polygenic risk scores for depressive symptoms significantly predicted suicidal ideation, and some predictive ability was found for suicide attempts; the polygenic risk scores explained a significant amount of variance in suicidal ideation (lowest *p* = 0.008, explained variance ranging from 0.10 to 0.16 %) and, less consistently, in suicide attempts (lowest *p* = 0.04, explained variance ranging from 0.12 to 0.23 %). Polygenic risk scores did not significantly predict NSSI. Results highlight that individuals genetically predisposed to depression are also more likely to experience suicidal ideation/behaviour, whereas we found no evidence that this is also the case for NSSI.

Intentional self-harm contravenes the fundamental drive of self-preservation. However, both suicidal and non-suicidal self-injurious (NSSI) behaviours (i.e., with and without the direct intention to die, respectively) are surprisingly common in the population. Almost 1 in 10 adults have ever thought about taking their own life, more than 1 in 20 have engaged in NSSI, and more than 1 in 40 have attempted suicide (Nock et al. 2008; Swannell et al. 2014). Moreover, self-harm is the eighth leading cause of death in the US (Rockett and Caine 2015). Recently, both a suicidal behaviour disorder and NSSI disorder were included for the first time in the DSM-5 as separate conditions for further study (American Psychiatric Association 2013).

Several studies indicate that being depressed dramatically increases the risk to engage in both non-suicidal (Glenn and Klonsky 2013; Hankin and Abela 2011; Hawton et al. 2013; Keenan et al. 2014; Nock et al. 2006; Prinstein et al. 2010; Selby et al. 2012) and suicidal self-injurious behaviours (Bernal et al. 2007; Brown et al. 2000; Casey et al. 2006; Fergusson et al. 2005; Hawton et al. 2013; Sokero et al. 2005; Thompson and Light 2011; Tidemalm et al. 2008). A meta-analysis of psychiatric disorders in patients presenting to hospital following self-harm, showed that about 50 % of individuals also suffered from depression (Hawton et al. 2013). In another study of more than 20,000 suicide cases, depression and borderline personality disorder were associated with the greatest increase in suicide risk (Qin 2011). The fact that depression is a major risk factor for self-injury may come as no surprise given that depression often includes symptoms such as hopelessness, negative affect, or recurrent thoughts of death and suicide (American Psychiatric Association 2013). Depression and self-injurious behaviours may be partly influenced by environmental factors, such as stressful life events (e.g., abuse/assault, interpersonal problems or employment difficulties; Haw and Hawton 2008; Kendler et al. 1999). However, genetic factors also contribute heavily to both self-injurious behaviours and depression. Twin studies indicate that between 30 and 60 % of the variance in non-suicidal and suicidal self-injurious behaviours and depression are attributable to genes (Durrett 2006; Kendler et al. 2006; Maciejewski et al. 2014; Sullivan et al. 2000; Voracek and Loibl 2007; Wray and Gottesman 2012). The identification of specific genetic variants for depression and self-injurious behaviours has so far achieved limited success (Galfalvy et al. 2011; Hek et al. 2013; Major Depressive Disorder Working Group of the Psychiatric 2013; Mullins et al. 2014; Schosser et al. 2011; Sokolowski et al. 2014; Willour et al. 2012), although some SNPs have been identified for major depression (Converge Consortium 2015) and depressive symptoms (Okbay et al. 2016).

As both traits are substantially heritable, the association between depression and self-injurious behaviours may be partly due to overlapping genetic risk. Twin studies have reported significant genetic correlations between suicidal ideation and depression (Linker et al. 2012), and between NSSI and internalizing disorders and suicide attempts and internalizing disorders (Durrett 2006). Advances in data availability (due to economically viable genome-wide genotyping) and methodology now allow an alternative means to directly test whether the genetic variance in depression also predicts an increased risk for self-injurious behaviours. Estimating the cumulative risk conferred by multiple risk alleles is referred to as polygenic risk scoring and has become increasingly popular for understanding both the genetic architecture of traits and the covariance between different traits (Dudbridge 2013).

Recently, a study has tested the association of polygenic risk scores for major depressive disorder with suicidal ideation and attempts in several target samples of mood disorder patients (Mullins et al. 2014). The study showed that polygenic risk scores for depression predicted suicidal ideation in a target sample of 747 individuals with up to 1 % explained variance. The prediction results for suicide attempts were inconsistent; the association did not reach significance in the three individual target datasets, and was significant only for two of the five subtests when combining the three datasets (up to 0.3 % explained variance; each subtest used different *p* value thresholds for inclusion of genetic risk variants for depression to create the polygenic risk scores). Findings suggest that depression and suicidal behaviours share some degree of genetic pleiotropy, but associations with suicide attempts were weak and replication may thus be warranted to strengthen the findings.

In this study we aim to replicate and extend the findings of Mullins et al. (2014) by performing polygenic risk scoring analyses to test whether the aggregated effects of common genetic variants underlying depression symptom count can predict suicidal ideation, suicide attempts and NSSI in a large population-based sample of 6237 individuals. Contrary to Mullins et al. (2014), whose polygenic risk scores were derived from the Psychiatric Genomics Consortium (PGC, *N* = 9240 mood disorder cases and 9519 controls; Major Depressive Disorder Working Group of the Psychiatric 2013), the polygenic risk scores for the present study were based on results from a genome-wide association study on depressive symptom count (*N* = 34,549; Hek et al. 2013). We used this continuous measure, because depression lies on a continuum and even sub-threshold depression is associated with increased suicidal risk (Ayuso-Mateos et al. 2010; Fergusson et al. 2005; Lewinsohn et al. 2000) and continuous measures—rather than clinical cut-offs—provide increased statistical power in genetic studies on psychopathology (van der Sluis et al. 2013). We used a considerably larger target sample for suicidal ideation than in the Mullins et al. study (2014; *N* = 6236 vs *N* = 747), providing greater power to accurately estimate genetic prediction. Importantly, this is the first study to estimate whether a genetic predisposition to depressive symptoms is associated with increased prevalence of NSSI.

## Methods

### Sample

The target sample consisted of twins and their family members from the Australian Twin Registry, a population-based twin registry. Between 1992 and 2009 these individuals participated in various semi-structured telephone interviews focused primarily on psychiatric disorders; details of the individual studies can be found elsewhere (Heath et al. 1999, 2011; Knopik et al. 2004). In all studies, the same items about NSSI, suicidal ideation, and suicide attempt were included.

Our final sample with both genotype and phenotype data comprised 6237 participants (2497 males and 3740 females) from 3473 families, including 2115 monozygotic and 2609 dizygotic twins and 1513 other family members. The participant’s age at the time of the survey ranged from 19 to 89 years (*M* = 42.40, *SD* = 11.67).

### Measures

Lifetime NSSI, suicidal ideation and suicide attempts were assessed as part of the SSAGA (Semi-Structured Assessment for the Genetics of Alcoholism), which assesses alcoholism and related disorders. The SSAGA has been shown to have good reliability and validity (Bucholz et al. 1994; Hesselbrock et al. 1999). The item used to determine lifetime NSSI was: “Did you ever hurt yourself on purpose, for example, by cutting or burning yourself?”^1^; the item used to determine suicidal ideation was: “Have you ever thought about taking your own life?” and the item used to determine suicide attempts was: “Have you ever tried to take your own life?”. All items were dichotomous (0 = *no*, 1 = *yes*). Complete data for NSSI, suicidal ideation, and suicide attempts were available for 4223, 6236, and 6226 individuals, respectively. Note that one cohort did not receive the question on NSSI, which accounts for the differences in sample size.

### Genotyping and quality control

DNA samples were collected in accordance with standard protocols and genotyped on various Illumina single nucleotide polymorphism (SNP) platforms (I317 K; I370 K-Duo; I370 K-Quad; I610 K-Quad; I660 K). Standard platform specific quality control procedures (described elsewhere; Medland et al. 2009) were applied before imputation, including checks for ancestry outliers, minor-allele frequency (MAF), Hardy–Weinberg Equilibrium (HWE), Mendelian errors and individual and SNP call rate. SNPs were then imputed using MACH v1 reference data from HapMap (Phases I and II, Release 22 Build 36). Subsequently, we performed a second round of quality control in which we deleted individuals with a call rate <95 % and all SNPs with an imputation quality of r^2^ < 0.30, MAF < 0.01, HWE test *p* value < 0.0001, or a call rate <95 %. We also checked for strand-flips and included only SNPs that were present in the summary statistics of the full meta-analysis.

### Generation of the polygenic scores and risk prediction analysis

Polygenic risk scores for each individual in the target sample were calculated in PLINK (Purcell et al. 2007) using the *p* values and *z*-scores (converted into betas) obtained from the summary statistics from the meta-analysis on depressive symptom count (Hek et al. 2013). The polygenic scores for individuals in the target sample were constructed by multiplying the number of copies of the effect allele at each SNP by the regression beta weight, and summing across SNPs. Scores were generated for nine different *p* value cut-offs for inclusion of risk variants for depressive symptoms (i.e P values of the association results for the SNPs): 0.001, 0.01, 0.05, 0.1, 0.2, 0.3, 0.4, 0.5 and 1.0. Before generating the polygenic scores, we identified ‘independent’ signals of association in the GWA meta-analysis results, using the linkage disequilibrium based “clumping” procedure as implemented in PLINK (Purcell et al. 2007). We used an LD threshold of r^2^ = 0.25 within a 250-kb window and the clumping procedure was repeated for all nine different risk prediction cut-offs (i.e. from *p* < 0.001 to *p* < 1.0), using the corresponding *p* value threshold (‘–clump-p1’). The number of SNPs that were retained for the generation of the risk scores ranged from *N* = 650 (for the significance threshold of *p* < 0.001) to *N* = 194,886 (for *p* = 1).

The risk prediction analysis was performed in SPSS version 23 using generalized estimation equations (GEE) with a logit link function. To account for relatedness of the sample, an exchangeable conditional covariance matrix was used (which allows for correlated residuals between members of the same family) and tests were based on the robust sandwich-corrected standard errors (Minică et al. 2015). We included sex, age, birth cohort (before 1951, 1951–1965, after 1965), and the first ten principal components of genetic variation (to correct for ancestry effects) as covariates in the analyses. Variance explained by the polygenic scores was calculated (in a logistic regression in SPSS) as the Nagelkerke’s pseudo-*R* ^2^ of the model including polygenic scores and all covariates minus the Nagelkerke’s pseudo-*R* ^2^ of the model including only covariates.

## Results

### Descriptive statistics

The overall prevalence for NSSI, suicidal ideation, and suicide attempts were 3.2, 27.1, and 4.0 %, respectively. Men and women did not differ on NSSI prevalence, χ^2^(1) = 0.01, *p* = 0.95. Men reported significantly more suicidal thoughts, 29.4 % versus 25.6 %, χ^2^(1) = 10.72, *p* = 0.001, whereas women reported significantly more suicide attempts, 4.5 versus 3.1 %, χ^2^(1) = 7.47, *p* = 0.006. Moreover, lifetime NSSI, suicidal ideation, and suicide attempts were all associated with significantly younger age, all *p*s < 0.003. The phi coefficients between NSSI and suicidal thoughts and attempts were φ = 0.19 and φ = 0.21 respectively, and φ = 0.33 between suicidal thoughts and suicide attempts (all *p*s < 0.001).

### Prediction

**Fig. 1:**
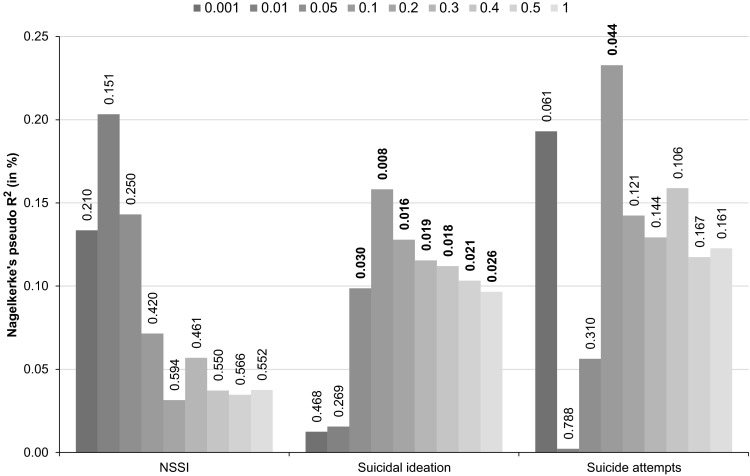
Results of polygenic risk score analysis. Variance explained (Nagelkerke’s pseudo R^2^) in NSSI, suicidal ideation, and suicide attempts by polygenic risk scores derived from depressive symptoms. Polygenic scores were created using nine significance thresholds for the inclusion of risk variants for depressive symptoms, ranging from *p* = 0.001 to *p* = 1.0. Values on the outside end of each bar denote the corresponding *p* value of the risk score prediction. *p* values at *p* < 0.05 are printed in bold

Results from the polygenic risk score analyses are shown in Fig. 1. The polygenic risk scores for depressive symptoms were significantly positively associated with suicidal ideation in our target sample for all significance thresholds from *p* < 0.05 upwards, with the risk scores explaining between 0.10 and 0.16 % of the variance in suicidal ideation. This indicates that individuals with a genetic predisposition to depression are also significantly more likely to experience suicidal ideation.

The risk scores also significantly predicted suicide attempts at the significance threshold of *p* < 0.1, for which the risk scores explained 0.23 % of the variance in suicide attempts. However, prediction did not reach significance for any of the other *p* value thresholds, even though the variance explained for suicide attempts was between 0.12 and 0.23 % (for the thresholds from *p* < 0.1 to *p* < 1.0), which is comparable to the variance explained in suicidal ideation. Depressive symptoms risk scores did not significantly predict variance in NSSI in our target sample under any of the significance thresholds, with estimates of variance explained ranging between 0.03 and 0.20 %.

## Discussion

Using a sample of 6,237 adults, we examined the extent to which polygenic scores for depressive symptoms were associated with an increased risk for suicidal ideation, suicide attempts, and NSSI. Polygenic scores for depressive symptoms consistently predicted suicidal ideation (from significance thresholds of *p* < 0.05 upwards for inclusion of risk variants for depressive symptoms). When including fewer SNPs from the depressive symptom count GWAS (significance threshold of *p* < 0.001 or *p* < 0.01), risk scores were not able to predict suicidal ideation or attempt, emphasizing the polygenic architecture of the traits. Some predictive ability was found for suicide attempts, although only one of the significance thresholds reached significance and thus results should be treated with caution. The genetic risk scores explained a comparable amount of variance in suicidal ideation (between 0.10 and 0.16 %) and suicide attempts (between 0.12 and 0.23 %; for significance thresholds of *p* < 0.1 upwards). However, our sample provided less power for suicide attempts, due to a lower number of cases. Importantly, we estimated for the first time whether a genetic predisposition to depressive symptoms is associated with increased prevalence of NSSI, and we found that polygenic risk scores did not predict NSSI. Altogether, our results suggest that individuals who are genetically vulnerable to depressive symptoms are at higher risk for suicidal thoughts and potentially suicide attempts, whereas no evidence was found for NSSI.

Our results for suicidal ideation and suicide attempt are consistent with findings from another recent study in which risk scores for major depressive disorder were found to be associated with suicidal ideation and attempts in patients with mood disorders, although the association with attempts only reached significance when combining the three target datasets (Mullins et al. 2014). Here we showed that the association with suicidal ideation (and to a lesser extent with suicide attempts) is also present in a population-based sample (as opposed to a sample selected for major depressive disorder) and using genetic risk scores for a continuous measure of depressive symptoms rather than a dichotomous measure of depressive disorder. Although GWA studies have not been very successful in identifying replicable genetic variants for major depressive disorder (Major Depressive Disorder Working Group of the Psychiatric 2013; although see Converge Consortium 2015) or depressive symptoms (Hek et al. 2013), we and others (Mullins et al. 2014) have shown that the aggregate effect of many common genetic variants underlying depression can explain a significant part of individuals’ liability to suicidal self-injurious thoughts and behaviours. This evidence provides additional support for current diagnostic criteria for major depressive disorder, which include suicidal ideation and behaviours.

We performed the first genetic risk prediction study for NSSI. Although twin studies have indicated a genetic overlap between NSSI and internalizing disorders (Durrett 2006), with the genetic risk prediction methodology, we did not find evidence that a genetic predisposition to depressive symptoms was associated with an increased risk for NSSI. Estimates of explained variance (between 0.03 and 0.07 % for significance thresholds of *p* < 0.1 upwards) are somewhat lower than the explained variance for suicide attempts and ideation. This may indicate a relatively lower genetic covariation between depressive symptoms and NSSI. Indeed, other studies have indicated that, on the phenotypic level, the association of depression with NSSI is somewhat lower than with suicidal behaviours (Claes et al. 2010; Csorba et al. 2009; Dougherty et al. 2009). Additionally, while our target sample was quite large (*N* = 4223), the sample was smaller than for the other two variables and the prevalence of NSSI was relatively low, so the absence of a significant association may also be the result of reduced power to detect small associations.

Some limitations need to be taken into account. First, the phenotypes were based on single-item questions, which may have introduced measurement error. For instance, with regard to NSSI, we did not have data on the types of NSSI and thus the category may contain also individuals which have engaged in less severe NSSI (e.g., single instances of hair pulling). Similarly, we did not differentiate between brief and sustained suicidal thoughts. These rather heterogeneous categories may therefore limit the power to detect polygenic effects. Indeed, the prevalence of suicidal ideation was relatively high in our sample (27.1 %), higher than in other studies (Nock et al. 2008). However, the prevalences of NSSI and suicide attempts (3.2 and 4.0 %, respectively) were comparable to other population studies in adults (Nock et al. 2008; Swannell et al. 2014). Second, although our risk scores can explain a significant amount of the vulnerability to suicidal self-injurious behaviours, the explained variance was very low, with the highest variance explained being 0.16 % for suicidal ideation and 0.23 % for suicide attempts. This is lower than in the study of Mullins et al. (2014), whose major depressive disorder risk scores explained up to 1 % of the variance in suicidal ideation and 0.3 % in suicide attempts. However, polygenic risk scores for depression can so far only explain up to 1 % variance in depression itself (Converge Consortium 2015; Demirkan et al. 2011; Major Depressive Disorder Working Group of the Psychiatric 2013; Peyrot et al. 2014). The accuracy in risk prediction studies depends largely on the size of the discovery sample from which SNP effects are used to create polygenic scores. Very large discovery samples are needed to accurately estimate single SNP effects (Wray et al. 2013), because summing the estimates of SNP effects also sums the error of those estimates, creating statistical noise. While we used the results from the largest GWAS meta-analysis of depression related phenotypes to date, larger samples will provide more accurate estimates of SNP effects leading to more accurate risk prediction. The Psychiatric Genomics Consortium is currently performing a second major depressive disorder GWA meta-analysis with a larger sample size; future studies using these results may be able to explain a larger part of the variation. Moreover, a recent study suggests that SNP effects for depression are more accurate when using a more homogenous depression measure. This study was the first to identify two loci of major depressive disorder, which the authors attributed to the inclusion of only homogenous cases with severe and recurrent depression episodes (Converge Consortium 2015). However, even a small amount of variance explained in a phenotype can have a critical influence on the development of a particular condition, especially if the genetic predisposition is a necessary precursor to the development of the condition.

Here, the combined effects of thousands of major depressive disorder SNPs only explained a small part of the genetic variation in self-injurious behaviours. It is possible that part of the genetic variation in self-injurious behaviours (and its genetic covariance with depression) is due to genetic variance not taken into account in the current risk-prediction methodology which only focusses on SNPs, i.e. common genetic variants. Future studies may also benefit from accounting for rare genetic variants and non-additive genetic effects. On the other hand, Lubke et al. (2012) showed that the vast majority of genetic variation underlying major depressive disorder could be attributed to common additive genetic effects.

Moreover, NSSI and suicidal ideation are likely to capture genetic liability to several traits in addition to depression. For instance, neuroticism (highly genetically correlated with depression, *r*_g_ = 0.75; Okbay et al. 2016), anxiety, aggression, impulsivity, psychosis, and schizophrenia may also play an important role in self-harm behaviours (Alaräisänen et al. 2009; Chioqueta and Stiles 2005; Dumais et al. 2005; Haw et al. 2001; Kiekens et al. 2015; Koyanagi et al. 2015; Mc Closkey et al. 2012; Sareen et al. 2005). Therefore, it would be interesting to investigate the genetic association between self-harm and other psychiatric disorders or traits in future risk prediction studies.

Further, environmental factors play a role in depression and self-injurious behaviours (Haw and Hawton 2008; Kendler et al. 1999). Thus, future studies could also examine how the environment moderates the genetic risk for depression to explain more variance in suicidal behaviours. A recent study found that the main effect of polygenic risk scores for depression as well as the interaction between the polygenic risk scores with the environment (childhood trauma) explained a comparable amount of variance in major depressive disorder (both 0.5 %; Peyrot et al. 2014).

In sum, the present study showed that a genetic predisposition to depressive symptoms plays a role in the pathogenesis of suicidal ideation, and possibly also in suicide attempts. We, however, did not find evidence that this is also the case for NSSI. Our results further emphasize the polygenic nature of complex psychiatric traits; like other mental disorders, self-injurious behaviours are likely to be due to the aggregate effect of many genetic variants, each of which on its own has a very small effect size. Future studies based on results from larger discovery samples, using larger target samples, and methodologies taking into account non-additive genetic effects and rare genetic variants as well as interactions between genes and environment may be able to further disentangle the (genetic) correlation between depression and self-injurious behaviours.

## Footnotes

1 If participants endorsed the suicide attempt items, then the NSSI item started with “Other than when you tried to take your own life”.
